# Effect of Cobalt–Chromium–Molybdenum Implant Surface Modifications on Biofilm Development of *S. aureus* and *S. epidermidis*

**DOI:** 10.3389/fcimb.2022.837124

**Published:** 2022-03-01

**Authors:** Astrid H. Paulitsch-Fuchs, Benjamin Bödendorfer, Lukas Wolrab, Nicole Eck, Nigel P. Dyer, Birgit Lohberger

**Affiliations:** ^1^Biomedical Sciences, University of Applied Sciences Carinthia, Klagenfurt, Austria; ^2^Diagnostic and Research Institute of Hygiene, Microbiology and Environmental Medicine, Medical University of Graz, Graz, Austria; ^3^Department of Orthopaedics and Trauma, Medical University of Graz, Graz, Austria; ^4^Bioinformatics Research Technology Platform, University of Warwick, Coventry, United Kingdom

**Keywords:** *Staphylococcus aureus*, *Staphylococcus epidermidis*, biofilms, CoCrMo, prosthetic infections

## Abstract

Periprosthetic infections are an eminent factor in patient care and also having significant economic implications. The number of biofilm-infection related replacement surgeries is increasing and will continue to do so in the following decades. To reduce both the health burden of the patients and the costs to the healthcare sector, new solutions for implant materials resistant to such infections are necessary. This study researches different surface modifications of cobalt–chromium–molybdenum (CoCrMo) based implant materials and their influence on the development of biofilms. Three smooth surfaces (CoCrMo, CoCrMo TiN, and CoCrMo polished) and three rough surfaces (CoCrMo porous coated, CoCrMo cpTi, and CoCrMo TCP) are compared. The most common infectious agents in periprosthetic infections are *Staphylococcus aureus* and Coagulase-negative *staphylococci* (e.g., *Staphylococcus epidermidis*), therefore strains of these two species have been chosen as model organisms. Biofilms were grown on material disks for 48 h and cell number, polysaccharide content, and protein contend of the biofilms were measured. Additionally, regulation of genes involved in early biofilm development (*S. aureus icaA*, *icaC*, *fnbA*, *fnbB*, *clfB*, *atl*; *S. epidermidis atlE*, *aap*) was detected using RT-q-PCR. All results were compared to the base alloy without modifications. The results show a correlation between the surface roughness and the protein and polysaccharide content of biofilm structures and also the gene expression of the biofilms grown on the different surface modifications. This is supported by the significantly different protein and polysaccharide contents of the biofilms associated with rough and smooth surface types. Additionally, early phase biofilm genes (particularly *icaA*, *icaC*, and *aap*) are statistically significantly downregulated compared to the control at 48 h on rough surfaces. CoCrMo TiN and polished CoCrMo were the two smooth surface modifications which performed best on the basis of low biofilm content.

## Introduction

Due to a general increase in life expectancy, improved surgical techniques and medical care, the demand for implants (e.g., joint replacement prosthesis) has increased greatly over recent decades. Although new materials or surface modifications for implants are constantly being developed, the struggle with periprosthetic infections is far from coming to an end. As an example, predictions for the US (compared to 2014) and Germany (compared to 2016) show an increase in primary total knee arthroplasty (TKA) by 2030 from 680,000 by 147% (Sloan et al., 2018) and from ~170,000 by between 8 and 49% (Rupp et al., 2020), respectively. Periprosthetic infections have an incidence of approximately 1–4% after primary TKA (Phillips et al., 2006). The incidence rate for a periprosthetic infection following a TKA replacement has been reported to be approximately 0.5% after 1 year, 0.8% after 5 years, and 1.4% after10 years (Tsaras et al., 2012), with cost per patient associated with TKA replacement of up to ~30,000 USD (Palsis et al., 2018).

Those periprosthetic infections can be caused by a number of different organisms, most of which are bacteria. In most cases a bacterial infection forms a biofilm on the surface of the implant which makes it even harder to treat. In a 2020 literature review on the topic of biofilms in periprosthetic infections Shoji and Chen (2020) reported a prevalence of *Staphylococcus aureus* in such infections of 21–43.6% followed by 20–31% Coagulase-negative *Staphylococcus*. Biofilm building *Staphylococcus* species have a large number of attributes allowing them to avoid host defenses and antibiotic treatments. The extracellular polymeric substances (EPS), also called extracellular matrix (ECM), build a physical barrier for transport of chemicals (Singh et al., 2010; Idrees et al., 2021) and immune-cells (Zimmerli et al., 1984) and hold the biofilm structure together. In addition, quorum sensing (Kavanaugh and Horswill, 2016; Kim et al., 2017), higher mutation frequencies (Ryder et al., 2012), and dormant cells (Venter et al., 2017; Lamret et al., 2020; Shoji and Chen, 2020) contribute to the pathogenicity of those strains.

Because treatment of already established biofilms is so difficult, the development of surfaces which are less favorable for the bacteria to attach to in the first place is an ongoing research topic. Cobalt–chromium–molybdenum (CoCrMo) based implants are regularly used not only for total joint replacement but also in dentistry (Chen and Thouas, 2015). The CoCrMo alloy has excellent biocompatibility and mechanical properties, which makes it the preferred material for knee and ankle replacements (Chen and Thouas, 2015). Physical and chemical surface modifications of metallic implant materials aim to improve their surface charge, wettability, topography and chemistry (Munir et al., 2020) in order to improve their osseointegration abilities and in the same time lessen the number of biofilm infections. These modifications can be achieved by mechanical treatment of the surfaces like polishing processes and numerous coating methods like plasma spraying, physical vapor deposition, cathodic arc deposition, and sintering (Munir et al., 2020). For this study five surface modifications have been applied to a casted CoCrMo base alloy: titanium nitride (TiN), mechanical polishing, porous coating, commercially pure titanium coating (cpTi), and a coating with tricalcium phosphate (TCP). Two bacterial species, *S. aureus* and *S. epidermidis*, were used as model organisms to monitor biofilm development after 48 h of incubation on the different alloy surfaces. Total cell count, protein and polysaccharide content of the biofilms were measured and data on biofilm associated gene expression was collected. The aim of the study is to understand the influence of the different CoCrMo surface modifications on the biofilm formation of *S. aureus* and *S. epidermidis*.

## Material and Methods

### CoCrMo Surface Modifications

All materials tested in this study were manufactured by Implantcast GmbH (Buxtehude, Germany) and were produced in a disc shape with a thickness of 1 mm and a diameter of 14 mm using a precision casting process. Gamma irradiation was used for sterilizing all materials described hereafter. Special coatings were produced and applied by DOT Ltd (Rostock, Germany). The CoCroMo casting alloy is composed of 28.5–29.5% Cr, 5.75–6.25% Mo, less than 1% each of Ni, Fe, C, Si, Mn, W, P, N, Al, Ti; and Co (~61–64%) making up the balance. This lies well within the specifications for this material given by the ISO 5832-3 for CoCrMo casting alloy for surgical implants (ISO 5832-3, 2016). Mechanical properties of the base CoCrMo alloy were tested according to ISO 6892-1 (2019) and are given as: tensile yield point R_p0.2_ ≥450 megapascal (MPa), tensile strength R_m_ ≥665 MPa and elongation at fracture A ≥8%. Titanium nitride (TiN) modified alloy surfaces show better properties in terms of biocompatibility, wettability, surface roughness, friction coefficient, corrosion resistance, minimized wear and increased temperature resistance; the TiN coating also leads to a reduced release of cobalt and chromium ions (Van Hove et al., 2015; Thomas et al., 2016). The coating of the discs was achieved by cathodic arc deposition. This technique is frequently used to synthesize extremely hard films for protecting the surfaces of materials. For the deposition a TiN target with ~99.4% titanium and less than 0.25% each of Fe, O, C, N and H (all according to ISO 5832-2 (2018)). The coating thickness was 5.5 ± 1.5 µm, the adhesive tensile strength ≥22 MPa and the layer roughness <0.05 µm. On top of the TiN layer an additional layer of ≥0.02 µm gold and cobalt (AuCo; with a maximum percentage of 0.2 ± 0.02% cobalt) was applied using a PVD-DC-Magnetron sputter. The density of the AuCo layer was 19.32 g/cm^3^, the specific electrical resistance was 2.35 µΩ • cm and the tensile strength is sufficient to prevent delamination of the coating when using an adhesive film strip-test. Highly polished CoCrMo alloys are commonly used, where the increased surface smoothness is associated with improved corrosion and wear properties (Davis, 2003). The porous coating was applied on the base material using sintering. In this process three layers of 250–355 µm diameter balls were applied on the material discs resulting in a coating thickness of 700–1,060 µm. Porosity of the coating was 30–40%, its tensile strength ≥34.5 MPa and its shear strength ≥20 MPa. The porous structure allows bone cells to “penetrate” into the implant, leading to a reduced rejection reaction. However, these pores also provide the bacterial cells with an increased surface area for adherence (Shoji and Chen, 2020; Idrees et al., 2021). The commercially pure titanium (cpTi) coating with a layer thickness of 300 ± 50 µm was sprayed onto the disc surfaces using a vacuum plasma spray (VPS). The resulting coating had a porosity of 30 ± 10%, an average roughness of 50 ± 15 µm, a tensile strength of ≥22 MPa and a shear strength of ≥20 MPa. cpTi covered materials show an improved osteogenic differentiation potential (Lohberger et al., 2020a), since the given porous upper layer in combination with the increase in surface energy offers the bone cells an optimal structure for adhesion (Geetha et al., 2009). Tricalcium phosphate coating (TCP, Bonit^®^) led to a deposited layer of 20 ± 10 µm thickness and to a tensile strength of ≥15 MPa (ISO 13779-2, 2018). TCP consist of 70% brushite (CaHPO_4_2•H_2_O) and 30% hydroxyapatite (Ca_5_(PO_4_)_3_OH). The calcium phosphate provides an advantage for osteoinduction to its surface as its bioactivity is highly similar to that of bone material and it thus facilitates improved cell growth and cytocompatibility (Dantas et al., 2018).

### Scanning Electron Microscopy (SEM)

SEM investigations were performed on a FEI Quanta 250 FEG (Thermo Fisher Scientific, Hillsboro, OR) under high vacuum conditions and 20 kV high tension. The micrographs were recorded in secondary electron (SE) mode with the Everhart–Thornley detector. The disc surfaces of the material were sputter coated with a gold layer (10 nm) to provide adequate electrical conductivity. The energy-dispersive X-ray spectroscopy (EDX) data collection measurements took 60 s each at 20 kV high tension and a Spotsize of 4.5 with a 30 mm² Octane Elect Plus Silicon Drift Detector (EDAX Ametek, NJ, USA) and the APEX Standard Software (V1.3.1, 07/2019) was used.

### Bacterial Cultures

For each experimental run, one overnight culture was prepared for each *S. aureus* subsp. *aureus* strain Newman D2C (ATCC 25904, Wesel, Germany; also referred to as NCTC 10833 or *S. aureus* subsp*. aureus* Rosenbach) and *S. epidermidis* (ATCC 14990, Wesel, Germany; also referred to as NCTC 11047). Luria–Bertani broth (LB broth) containing 10 g/L tryptone, 5 g/L yeast extract (both Carl Roth), and 5 g/L sodium chloride (Merck, Darmstadt, Germany) was used as growth medium. Per strain one CRYOBANK^®^ pearl (MAST Group, Reinfeld, Germany) was inoculated into 100 ml LB and cultures were incubated at 37°C at 90 rpm.

### Biofilm Assay

**Figure 1:**
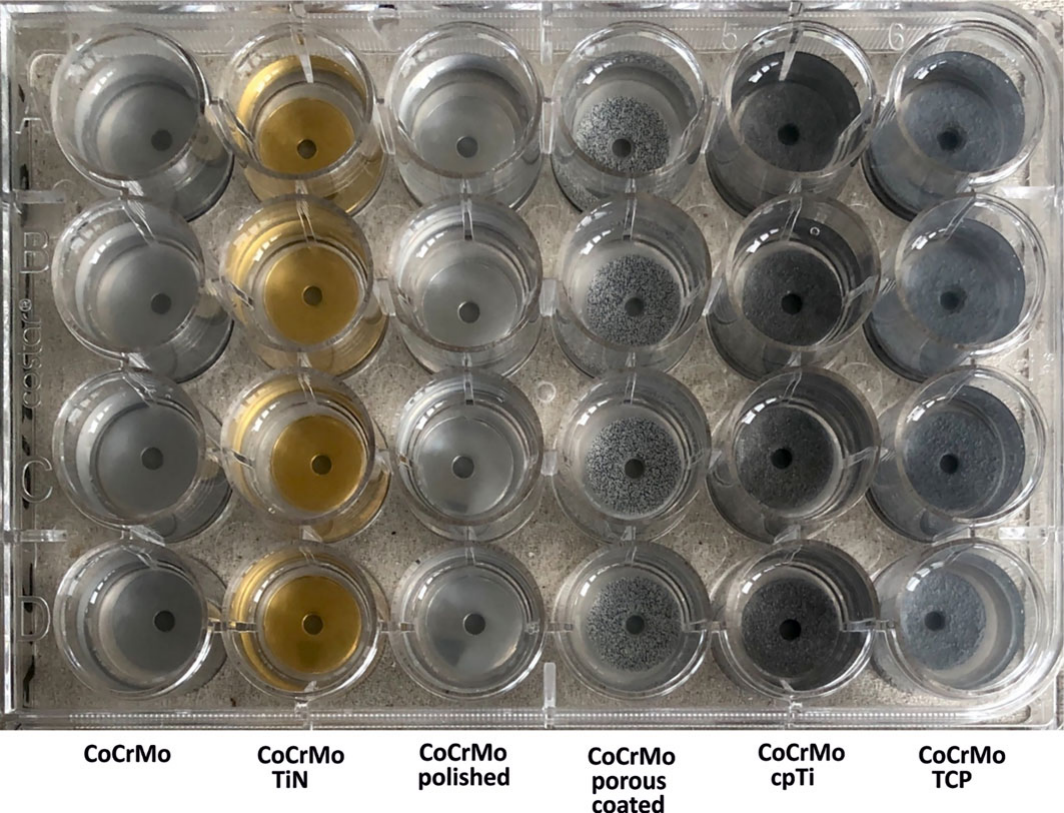
Cell culture plate with the different alloy discs.

The material discs (4 discs per material, all 6 materials) were placed in 24-well untreated clear polystyrene plates (Corning^®^, Wiesbaden, Germany) as shown in **Figure 1**. The bacterial cells from the overnight culture were distributed into 1.5 ml Eppendorf tubes and collected by centrifugation (14,000 rpm, 2 min). The supernatant was discarded and the cells where resuspended and washed in 1.5 ml of phosphate buffered solution (PBS). Cells were centrifuged again (14,000 rpm, 2 min) and then freshly inoculated into LB broth. The cell number was adjusted to 1.5 × 10^8^ CFU/ml in LB broth and 1.5 ml of the adjusted cell solution was added to each well. The plate was then sealed with a Breathe Easy^®^ sealing membrane (Merck, Darmstadt, Germany) and incubated for 48 h at 37°C and 90 rpm. A total of 4 discs per material and species were prepared for each of the 21 experimental runs (biological replicates). Growth controls (bacteria without discs) and sterile controls (sterile LB media on material discs) were run in parallel for every experimental run.

### Biofilm Processing

For the collection of the samples the medium was first gently removed without disturbing the biofilm and 1.5 ml PBS was added into each well. The discs were picked up using tweezers, shaken within the PBS and the biofilm from all sides of the disk was scraped into a fresh PBS filled well with a mini cell scraper (Biotium, Freemont, CA, USA). At the end of the process the scraper was vigorously rotated in the well to ensure that no biofilm residues remained on the scraper. The content of two wells per material was pooled in a 3.5 ml tube and vortexed until no biofilm parts were visible. This step took up to 30 min on the vortexer in continuous mode and random samples were checked microscopically to ensure that there were no more biofilm parts in the samples before further processing. The growth controls were re-suspended in the medium and then transferred into 3.5 ml tubes; the sterile controls were also pipetted into the individual collection tubes directly. The resulting 3 ml sample volume (per biofilm pool and controls) was divided to provide the volumes needed for the four different measurement protocols (see below). Samples for genetic analysis were frozen at −80°C until further processing, all protein and polysaccharide samples were stored in the fridge at 4°C for no longer than 24 h before analysis, flow cytometry was performed directly after biofilm processing and samples were kept at 4°C until loading onto the instrument.

### Polysaccharide Quantification

Polysaccharides of the total biofilm were quantified using an adapted version of an sulfuric acid phenol extraction method (Cuesta et al., 2003). Approximately 250 µl of each sample, 250 µl 99.5% phenol, and 750 µl 95–98% sulfuric acid were added to heat-resistant glass tubes, sealed with aluminum foil (not air tight) and vortexed for at least 20 s. Incubation took place in a water bath at 100°C for 10 min. In an additional water bath samples were cooled to 25°C, vortexed again and 250 µl was transferred into uncoated U-bottom 96-well plates (BRAND^®^, Sigma-Aldrich, Darmstadt, Germany). Absorbance was read at 490 nm in the Multiskan Sky Microplate Spectrophotometer (Thermo Fisher Scientific). Each sample was measured twice and the statistical mean was compared to a standard glucose curve (Merck; 0–1.5 µg/ml).

### Protein Quantification

The **‘**Pierce ™ BCA Protein Assay Kit**’** (Thermo Fisher Scientific, Waltham, MA, USA) was used to measure the total protein content of the biofilms. The 562 nm absorbance values of the samples (25 µl sample in 200 µl working reagent from the kit) in uncoated U-bottom 96-well plate were read on a Multiskan Sky Microplate Spectrophotometer. Each sample was measured twice. The standard curve was prepared with bovine serum albumin (BSA, supplied with the BCA kit; 0–2,000 µg/ml, Thermo Fisher Scientific, Waltham, MA, USA) and the arithmetic mean of the duplicate measurement was compared to the curve.

### Live Dead Assay

Flow cytometric cell counts were performed applying the ‘LIVE/DEAD^®^ BacLightTM Viability Kit (Invitrogen, Carlsbad, CA, USA) for microscopy and quantitative assays’. The Syto9^®^ and propidium iodide dye mixes were freshly prepared for each measurement in a ratio of 1:1. Per sample, 1 ml was stained with 1 µl of the dye mixture and incubated in the dark for 15 min at room temperature and 100 µl per sample were analyzed on a Cyflow^®^ Cube 6 flow cytometer (Sysmex Europe GmbH, Norderstedt, Germany). The flow rate was set at 2 µl/s and a 488 nm laser was used. All samples were measured twice and to avoid signal carryover, cleaning was performed between all measurements.

### Statistics

SPSS (IBM, version 25) was used for statistical analyses of protein, polysaccharide, and flow cytometry data. The data was found to be non-Gaussian (Kolmogorov–Smirnov test with Lilliefors correction). Consequently, the Kruskal–Wallis H test was applied. Statistical differences were tested in a pairwise comparison format and the Bonferroni correction for the Kruskal–Wallis test was used.

### RNA Isolation

RNA from the samples of three independent experimental runs (3 biological replicates) was extracted with the Monarch^®^ Total RNA Miniprep Kit (New England BioLabs, Ipswich, MA, USA). The enzymatic approach step of the manufacturer’s protocol was adapted: additionally, 0.1 mg/ml lysostaphin (Sigma-Aldrich, Darmstadt, Germany) was added to the 3 mg/ml lysozyme which was provided with the kit. Samples were incubated for 25 min at 350 rpm at 37°C before carrying out the rest of the protocol according to the guidelines. Final elution volume was 30 µl per sample.

### RT-qPCR

Using the iScript cDNA Synthesis Kit (BioRad Laboratories Inc., Veenendal, The Netherlands) 1 µg RNA was reverse-transcribed with a mixture of oligo (dT) and random hexamer primers. The samples were amplified with the SsoAdvanced Universal SYBR Green Supermix and subsequently measured on a CFX96 Touch (BioRad Laboratories Inc.), as described elsewhere (Lohberger et al., 2020b). A standard 3-step PCR temperature protocol was used with an annealing temperature of 60°C followed by a melting curve protocol to confirm a single gene-specific peak and to detect primer dimerization. The ΔΔCt method was applied for the calculation of the relative quantification of expression levels by means of the geometric mean of the internal control (16s rRNA gene for *S. aureus* and also for *S. epidermidis*; for primer sequences see **Table 1**). The expression levels (Ct) of the target genes were normalized to the reference genes (ΔCt). The ΔΔCt value was calculated using the difference between the ΔCt value of the test sample and the ΔCt of the control sample. The final expression ratio was expressed as 2ΔΔCt (Livak and Schmittgen, 2001). Primers used for RT-qPCR were purchased from Eurofins Genomics (Ebersberg, Germany) and primer sequences are listed in **Table 1**.

**Table 1:** Primes used in RT-q-PCR.

Strain	Gene	Primer forward	Primer reverse	Reference
*S. aureus*	*icaA*	5-GAGGTAAAGCCAACGCACTC-3	5-CCTGTAACCGCACCAAGTTT-3	Atshan et al. (2013)
	*icaC*	5-CTTGGGTATTTGCACGCATT-3	5-GCAATATCATGCCGACACCT-3	Atshan et al. (2013)
	*fnbA*	5-AAATTGGGAGCAGCATCAGT-3	5-GCAGCTGAATTCCCATTTTC-3	Atshan et al. (2013))
	*fnbB*	5-ACGCTCAAGGCGACGGCAAAG-3	5-ACCTTCTGCATGACCTTCTGCACCT-3	Atshan et al. (2013)
	*clfB*	5-AACTCCAGGGCCGCCGGTTG-3	5-CCTGAGTCGCTGTCTGAGCCTGAG-3	Atshan et al. (2013)
	*atl*	5-TTTGGTTTCCAGAGCCAGAC-3	5-TTGGGTTAAAGAAGGCGATG-3	Yin et al. (2018)
	*16S rRNA*	5´-GGGACCCGCACAAGCGGTGG-3´	5´-GGGTTGCGCTCGTTGCGGGA-3´	Atshan et al. (2013)
*S. epidermidis*	*atlE*	5-TGTCCTGCTTTCACGTATGA-3	3-TCTTTGGAATTGGTGCATTT-5	Patel et al. (2012)
	*aap*	5-TGATCGGATCTCCATCAACT-3	3-AAGGTAGCCAAGAGGACGTT-5	Patel et al. (2012)
	*16S rRNA*	5´TACACACCGCCCGTCACA	5´CTTCGACGGGCTAGCTCCAAAT	Vandecasteele et al. (2001)

## Results

### Material Surface Characteristics

**Figure 2:**
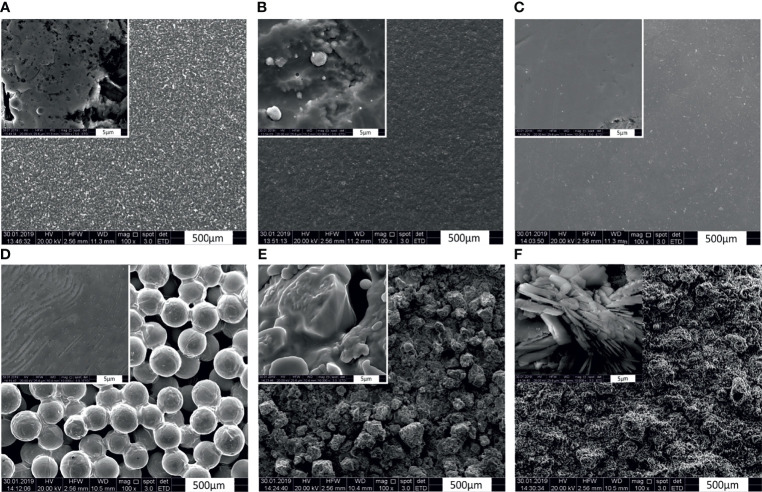
Scanning electron microphotographs of CoCrMo and the different surface modifications. Magnification factor ×100, and inlays ×10,000. **(A)** CoCrMo; **(B)** CoCrMo TiN; **(C)** CoCrMo polished; **(D)** CoCrMo porous coated; **(E)** CoCrMo cpTi, **(F)** CoCrMo TCP.

The surface characteristics of the different modifications to the CoCrMo alloy discs have been studied using both SEM and EDX analyses. The topographic characteristics of materials are well known to have a substantial influence on the adhesive properties of bacterial cells and therefore the microscopic investigation is helpful for understanding the data collected in this study. While the base alloy CoCrMo without modifications shows a rather smooth surface at ×100 magnification (**Figure 2A**), a closer look (×10,000, **Figure 2A** inlay) reveals only some cracks and some unevenness and the surface modification with TiN and the polished surface are even smoother. However, the porous coated surface and the cpTi and TCP covered surfaces show very distinct topographic characteristics. While the porous coated surface consists of a thick layer of evenly distributed (**Figure 2D**) and rather smooth balls (surface of the ball in the inlay of **Figure 2D**), the cpTi layer is rougher (**Figure 2E**) and the higher magnification also reveals that this characteristic is also true at the small µm range (inlay **Figure 2E**). Very distinctly different from the rest, the TCP layer forms sharp crystalline structures protruding from the alloy surface (**Figure 2F**). Looking at the surface characteristics, the surfaces can be categorized as either smooth (CoCrMo, CoCrMo TiN, and CoCrMo polished) or rough (CoCrMo porous coated, CoCrMo cpTi, and CoCrMo TCP).

**Figure 3:**
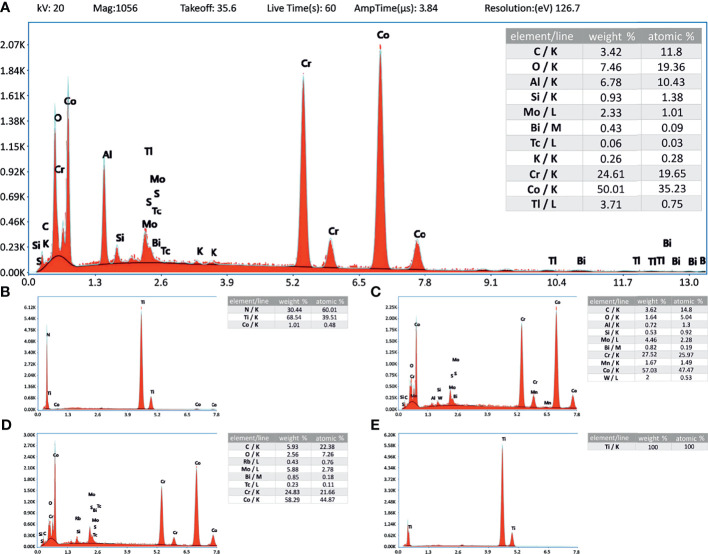
Energy-dispersive X-ray analysis. Inlay tables showing the elemental composition related to the energy level (K-, L- and M-line). Instrument values are given in panel **(A)** and apply also to **(B**–**E)**. **(A)** CoCrMo; **(B)** CoCrMo TiN; **(C)** CoCrMo polished; **(D)** CoCrMo porous coated; **(E)** CoCrMo cpTi.

The corresponding EDX data supports the material descriptions given in the material and methods section, showing the elemental composition of the materials as spectrograms and in weight and atom percentages (**Figures 3A**–**E**; data not available for TCP).

### Polysaccharide Content

**Figure 4:**
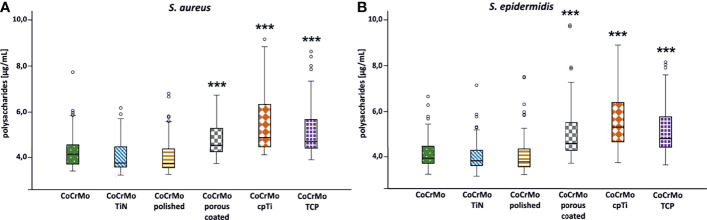
Polysaccharide concentration of *S. aureus* (**A**; n = 42) and *S. epidermidis* (**B**; n = 44) biofilms. Statistically significant differences to CoCrMo are marked *** (adjusted significance < 0.001, Bonferroni correction for the Kruskal–Wallis test).

As all *Staphylococci* can produce polysaccharides in their EPS when forming biofilms (Arciola et al., 2015), an approach that is often applied is to break the polysaccharides down into monosaccharides and measure the content compared to a glucose standard curve (Cuesta et al., 2003). Following this procedure, we found (**Table 2** and **Figure 4**) that the biofilms grown on the smooth surfaces (CoCrMo, CoCrMo TiN, and CoCrMo polished) show a tendency to form less polysaccharide in their EPS compared to those grown on the rough surfaces (CoCrMo porous coated, CoCrMo cpTi, and CoCrMo TCP). The highest polysaccharide levels for *S. aureus* were measured in biofilms grown on CoCrMo cpTi discs with a mean value of 5.69 ± 1.5 µg/ml, the lowest values were measured in biofilms from the CoCrMo TiN and the CoCrMo polished discs with 4.15 ± 0.71 µg/ml and ±0.8 µg/ml respectively (**Table 2**). *S. epidermidis* biofilms showed the highest polysaccharide values on CoCrMo cpTi (6.21 ± 3.06 µg/ml) and the lowest ones on CoCrMo TiN (4.11 ± 0.78 µg/ml) (**Table 2**). In both species the polysaccharide content of the biofilms grown on CoCrMo porous coated, CoCrMo cpTi and CoCrMo TCP shows a highly significant difference (p <0.001) compared to that of the CoCrMo alloy itself (**Figure 4**). **Table 3** summarizes all group comparisons (each alloy compared to each) where a clear statistical difference can be seen whenever a smooth surface is compared to a rough surface. One exception here is the comparison of *S. epidermidis* biofilms on CoCrMo porous coated with biofilms from CoCrMo cpTi surfaces where, in the overall group comparison (adjusted p-value for multiple comparisons), no statistical difference is found (adj. p-value = 0.055). However, if the multiple comparison is disregarded, which is possible in a direct comparison of the two groups in question, the difference becomes significant again (p = 0.004). This leads to the conclusion that the CoCrMo porous coated surface is the best performer (smallest biofilm content) in the rough surface group. Additionally, in the direct comparison of CoCrMo with CoCrMo polished (p = 0.006), a statistically significant difference is also detected, showing a better performance (less polysaccharides) compared with biofilms grown on the polished CoCrMo surface (again no significant difference in the adj. p-value = 0.086). Overall, CoCrMo polished performs the best with respect to polysaccharide formation of biofilms.

**Table 2:** Mean, minimum and maximum values and standard deviations (SD; 95% confidence interval) of protein and polysaccharide measurements.

Alloy	*S. aureus*				*S. epidermidis*			
	Proteins [µg/ml]		Polysaccharides [µg/ml]		Proteins [µg/ml]		Polysaccharides [µg/ml]	
	mean [min; max] (n = 42)	SD	mean [min; max] (n = 44)	SD	mean [min; max] (n = 42)	SD	mean [min; max] (n = 44)	SD
CoCrMo	124.56 [68.10; 214.10]	34.89	4.46 [3.47; 7.74]	0.91	101.95 [36.5; 203.5]	34.78	4.23 [3.25; 6.47]	0.75
CoCrMo TiN	97.55 [55.40; 148.60]	28.12	4.15 [3.27; 5.82]	0.71	77.65 [26.7; 162.4]	32.12	4.11 [3.15; 7.13]	0.78
CoCrMo polished	96.57 [41.80; 179.40]	30.8	4.15 [3.3; 6.75]	0.8	76.43 [31; 145.6]	30.22	4.12 [3.25; 7;50]	0.87
CoCrMo porous coated	225.33 [64.40; 301.70]	41.88	4.89 [3.77; 6.73]	0.78	207.32 [149; 275.5]	35.68	5.59 [3.77; 29.57]	3.88
CoCrMo cpTi	202.87 [121.20; 269.70]	35.5	5.69 [4.17; 9.01]	1.5	190.23 [115.1; 341.1]	42.79	6.21 [3.76; 22.69]	3.06
CoCrMo TCP	240.08 [153.10; 455.60]	58.6	5.24 [3.95; 8.53]	1.14	215.51 [113.3; 356.6]	56.42	5.27 [3.67; 8.51]	1.19

**Table 3:** Statistical pairwise comparison of alloys according to the protein and polysaccharide content of the *S. aureus* and *S. epidermidis* biofilms.

Alloy 1	Alloy 2	*S. aureus*				*S. epidermidis*			
		Proteins		Polysaccharides		Proteins		Polysaccharides	
		p-value	adj. p-value	p-value	adj. p-value	p-value	adj. p-value	p-value	adj. p-value
CoCrMo	CoCrMo TiN	0.005	0.074	0.036	0.539	0.011	0.162	0.255	1.000
CoCrMo	CoCrMo polished	0.004	0.058	0.012	0.178	0.006	0.086	0.212	1.000
CoCrMo	CoCrMo porous coated	0.000	0.000	0.000	0.002	0.000	0.000	0.000	0.000
CoCrMo	CoCrMo cpTi	0.000	0.000	0.000	0.000	0.000	0.000	0.000	0.000
CoCrMo	CoCrMo TCP	0.000	0.000	0.000	0.005	0.000	0.000	0.000	0.000
CoCrMo TiN	CoCrMo polished	0.939	1.000	0.675	1.000	0.850	1.000	0.912	1.000
CoCrMo TiN	CoCrMo porous coated	0.000	0.000	0.000	0.001	0.000	0.000	0.000	0.000
CoCrMo TiN	CoCrMo cpTi	0.000	0.000	0.000	0.000	0.000	0.000	0.000	0.000
CoCrMo TiN	CoCrMo TCP	0.000	0.000	0.000	0.000	0.000	0.000	0.000	0.000
CoCrMo polished	CoCrMo porous coated	0.000	0.000	0.000	0.000	0.000	0.000	0.000	0.000
CoCrMo polished	CoCrMo cpTi	0.000	0.000	0.000	0.000	0.000	0.000	0.000	0.000
CoCrMo polished	CoCrMo TCP	0.000	0.000	0.000	0.000	0.000	0.000	0.000	0.000
CoCrMo porous coated	CoCrMo cpTi	0.042	0.632	0.006	0.086	0.076	1.000	0.004	0.055
CoCrMo porous coated	CoCrMo TCP	0.806	1.000	0.199	1.000	0.995	1.000	0.270	1.000
CoCrMo cpTi	CoCrMo TCP	0.023	0.341	0.139	1.000	0.077	1.000	0.072	1.000

P-values (Kruskal–Wallis test); adjusted p-values (Bonferroni correction for the Kruskal–Wallis test of multiple comparisons; α = 5%); smooth surfaces (CoCrMo, CoCrMo TiN, CoCrMo polished) underlaid in light blue; rough surfaces (CoCrMo porous coated, CoCrMo cpTi, CoCrMo TCP) underlaid in gray; p < 0.05 also marked in green.

### Protein Content

**Figure 5:**
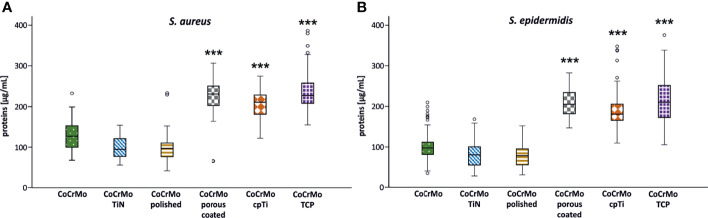
Protein concentration of *S. aureus* (**A**; n = 42) and *S. epidermidis* (**B**; n = 44) biofilms. Statistically significant differences to CoCrMo are marked *** (adjusted significance < 0.001, Bonferroni correction for the Kruskal–Wallis test).

Proteins in the bacterial EPS function as glue which sticks the biofilms to the surface. Measurements of proteins often employ colorimetric methods using the reduction of Cu^2+^ to Cu^1+^ copper ions in alkaline medium and compare the protein content to a bovine serum albumin (BSA) standard. Using a variation of this method (Pierce™ BCA Protein Assay Kit) we found that the highest protein levels in *S. aureus* occurred in biofilms (namely, EPS and cells) grown on CoCrMo TCP discs with a mean value of 240.08 ± 58.6 µg/ml. The lowest values for *S. aureus* were measured in biofilms from the CoCrMo polished discs with 96.57 ± 30.8 µg/ml (**Table 2**). *S. epidermidis* biofilms showed the highest polysaccharide values on CoCrMo TCP as well (215.51 ± 56.42 µg/ml) and the lowest ones on CoCrMo polished (76.43 ± 30.22 µg/ml) (**Table 2**). All *S. aureus* and all *S. epidermidis* biofilms from rough surfaces (CoCrMo porous coated, CoCrMo cpTi, CoCrMo TCP) have statistically significantly higher protein values (p <0.001) when compared to the base alloy (**Figure 5**). Again, all smooth surfaces have a statistically significantly better outcome compared to the rough surfaces (**Table 3**) when comparing them against each other separately. Additionally, in the case of *S. aureus*, CoCrMo polished and CoCrMo TiN perform statistically significantly better than CoCrMo (p = 0.004 and p = 0.005; meaning lower protein content) in the single comparison of groups (this significance however is not applicable for the multiple comparison; p = 0.058), leading overall to the best performance of CoCrMo polished. For the direct group comparison of *S. epidermidis* biofilm on CoCrMo compared to CoCrMo polished (less protein) this also applies (p = 0.006; adj p = 0.086). When considering the cpTi rough surfaces and *S. aureus*, CoCrMo cpTi results in less biofilm protein compared to CoCrMo TCP (p = 0.023; adj p = 0.341).

### Flow Cytometric Cell Enumeration

**Figure 6:**
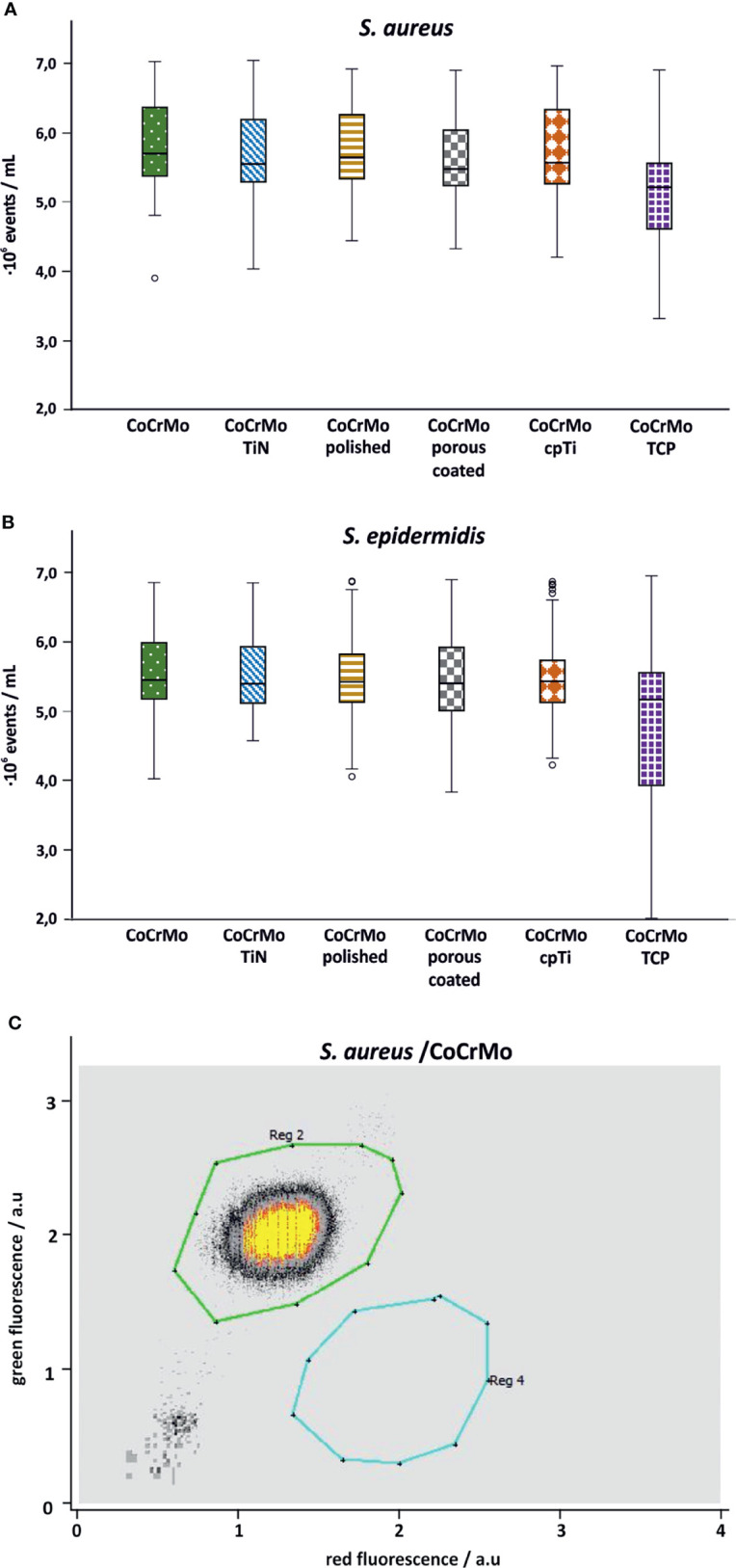
Flow cytometry cell counts for *S. aureus* (**A**; n = 42) and *S. epidermidis* (**B**; n = 44). Subpanel **(C)** shows a representative measurement of *S. aureus* on CoCrMo (Reg 2: living cells; Reg 4: dead cells).

Flow cytometric measurements on the samples were meant to show a live-dead count. However, it seems that within 48 h of growth, the dead cell count is negligible which is why the flow cytometry data are only used for cell enumeration. For both species no statistical differences in the number of bacterial cells have been detected (**Figure 6**).

### RT-qPCR

**Figure 7:**
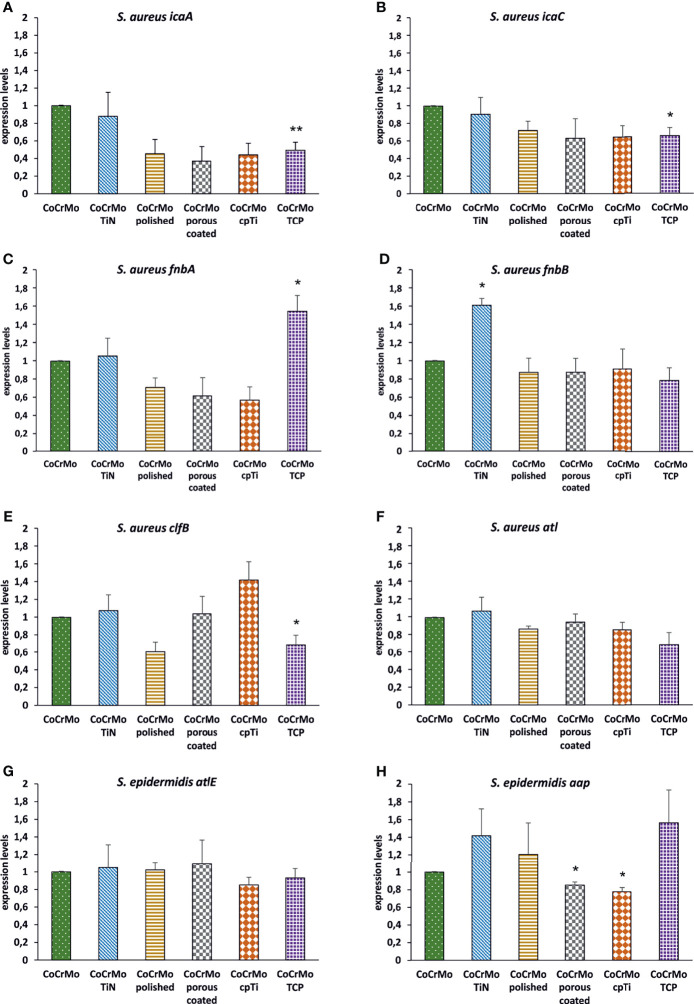
Expression levels of biofilm-associated genes. *S. aureus* (**A**–**F**; n = 3) and *S. epidermidis* (**G**, **H**; n = 3); error bars show the standard error of the mean; statistically significant differences to CoCrMo are marked * (p <0.05).

Many genes are involved in the biofilm development of *S. aureus* and *S. epidermidis*. A few of them have been chosen to understand the genetic reaction of both species to the different CoCrMo surfaces. Again, the different surface modifications were statistically compared to the base alloy in terms of gene expression levels of the biofilms harvested after 48 h of development (**Figure 7**). For *S. aureus*, *icaA*, and *icaC* were selected from the intercellular adhesion group of genes (*ica*) which is of specific interest for the starting phase in biofilm development. Only CoCrMo TCP showed a significantly decreased level of gene expression for both genes (**Figures 7A, B**). Looking at the fibronectin binding protein *fnb*A gene, our data also shows a significantly increased level for CoCrMo TCP biofilms. However, the closely connected *fnb*B was significantly elevated only in CoCrMo TiN. The *clf*B (bacterial ligand clumping factor) gene of *S. aureus* was, with p <0.05, decreased in CoCrMo TCP, whereas the major autolysine gene (*atl*) showed no significant changes in any of the surface modification biofilms of *S. aureus*. Of the two genes tested for *S. epidermidis*, the major autolysine (*atlE*) also did not show any differences. However, the expression of the *aap* (accumulation association protein) gene was significantly reduced in CoCrMo porous coated and CoCrMo cpTi, but interestingly, not in the third rough surface modification CoCrMo TCP which showed elevated levels (not significant).

## Discussion

Biofilm evaluation begins with the organisms chosen for the experiment. *S. aureus* and *S. epidermidis* are the most common causes of periprosthetic infections and have therefor been the model organisms for this study. But also, within the *S. aureus* and *S. epidermidis* isolates, differences in biofilm forming abilities exist, making it difficult to generalize results. *S. aureus* Newman has already been used for many studies on biofilm formation (e.g., Johnson et al., 2008; Abraham and Jefferson, 2012; Forson et al., 2020; Inés Molina et al., 2020; Pinto et al., 2020) although it is not considered a very good biofilm forming strain and the same is true for the *ica* negative *S. epidermidis* strain (e.g., Stepanovic et al., 2000; Lee et al., 2016; Paduszynska et al., 2019; Di Pilato et al., 2020; Paulitsch-Fuchs et al., 2021). However, the variant of the *S. aureus* strain used in this study *S. aureus* Newman D2C is considered to be a relatively good biofilm forming strain (Grundmeier et al., 2004; Tsompanidou et al., 2010; Abraham and Jefferson, 2012; Dauros-Singorenko et al., 2020; Paulitsch-Fuchs et al., 2021). Abraham and Jefferson (2012) showed that the autolysin activity in the Newman D2C variant was low enough to allow the expression of ClfB on the cell surface, which seems to be (at least partly) responsible for the difference in biofilm forming abilities between the two strains. The issue about different *S. aureus* Newman strains deposited to the reference centers with similar names and therefore leading to seemingly controversial results has also already been pointed out, e.g., by Grundmeier et al. (2004). It also has been shown previously (Dauros-Singorenko et al., 2020) that the D2C Newman strain expresses the *agr* (accessory gene regulation) quorum sensing system, which would normally mean that the dispersion in biofilms takes place rather easily (Paulander et al., 2018). However the LB medium we used is iron-rich (Abdul-Tehrani et al., 1999) and high iron contents did show a negative influence on *agr* expression in the hemoglobin study by Dauros-Singorenko et al. (2020), therefore possibly promoting biofilm formation of *S. aureus* Newman D2C in LB medium. This leads to another main influence on biofilm growth which is the medium used for the experiments. Several studies compared different growth media and their influence on the biofilm formation of *S. aureus* and other species (Del mar Cendra et al., 2019; Wijesinghe et al., 2019; Zhou et al., 2019; Liu et al., 2020). For example, in one study LB medium showed the second best performance for biofilm formation in *Pseudomonas aeruginosa* and *S. aureus* (Zhou et al., 2019), in another study (Del mar Cendra et al., 2019) LB medium also promoted the biofilm growth for *S. aureus* Newman (although the wildtype strain was used). A variety of differences exist also for LB medium recipes (e.g., supplemented with glucose, higher or lower NaCl content) and most other growth media. Lastly also the number of bacterial cells inoculated for biofilm formation varies greatly in the different studies on this topic. As little as OD_550_ of 0.10 (~0.015 × 10^7^ cells/ml) (Del mar Cendra et al., 2019) and also higher cell numbers of 1 × 10^7^ CFU/ml (Dauros-Singorenko et al., 2020) and inoculum sizes using 0.5 MacFarland cell suspensions (1.5 × 10^8^ cells/ml) (Wijesinghe et al., 2019) are implemented for biofilm studies. Therefore, the choice of the strains, inoculum size and media used in biofilm studies has to be well thought of.

Although the interaction of biofilms with the respected surfaces they grown on is a key factor as well, still understanding is lacking in some areas of this interaction, e.g., the influence of surface roughness on biofilm development and its connection to the regulation of genes connected to biofilm formation. For an overview and to facilitate this connection, **Table 4** provides an overall comparison of protein, polysaccharide and gene expression results.

**Table 4:** Overall comparison of the results of protein, polysaccharide and genetic measurements.

All values compared to CoCrMo	*S. aureus*								*S. epidermidis*			
	Proteins	Glucose	*icaA*	*icaC*	*fnbA*	*fnbB*	*clfB*	*atl*	Proteins	Glucose	*atlE*	*aap*
CoCrMo TiN	–	–	–	–	–	↑	–	–	–	–	–	–
CoCrMo polished	–	–	–	–	–	–	–	–	–	–	–	–
CoCrMo porous coated	↑↑↑	–	–	–	–	–	–	–	↑	↑↑↑	–	↓
CoCrMo cpTi	↑↑↑	↑↑↑	–	–	–	–	–	–	↑↑↑	↑↑↑	–	↓
CoCrMo TCP	↑↑↑	–	↓↓	↓	–	–	–	–	↑↑↑	↑↑↑	–	–

**↑**, **↑↑** and ↑**↑↑** (also underlaid in green; adjusted significance <0.05, <0.01 and 0.001 according to the Bonferroni correction for the Kruskal–Wallis test) and ↓, and ↓↓ (also underlaid in orange; adjusted significance <0.05 and <0.01 according to the Bonferroni correction for the Kruskal–Wallis test).

The surfaces studied here vary widely in their surface characteristics and structure (see **Figure 2**), thus providing a good variety of roughness for biofilm formation observations. Both protein and polysaccharide measurements are valuable measurements for the understanding of bacterial adherence and biofilm compactness in later stages of biofilm development. While host-proteins play an important role in the early conditioning of the implant thus strengthening the incorporation of the implant (Wang et al., 2017), proteins of biofilm organisms use the same properties to attach to the material in early biofilm development. Polysaccharides are important surface characteristics of bacterial cells and contribute to EPS development and to the attachment of bacterial cells to surfaces and to each other (Limoli et al., 2015). We show that the surface modifications resulting in rough surfaces (CoCrMo porous coated, CoCrMo cpTi, and CoCrMo TCP) have a higher polysaccharide and protein load in the biofilms than the smooth surfaces (CoCrMo TiN, CoCrMo polished) and the untreated control (CoCrMo). In most cases this is a statistically significant difference and holds for most of the comparisons of the alloys to each other as well (see **Table 3**). Those results are in accordance with earlier studies on the topic (Öztürk et al., 2007; Yoda et al., 2014; Kunrath et al., 2020; Palka et al., 2020; Paulitsch-Fuchs et al., 2021), supporting the conclusion that the higher surface roughness is beneficial for protein and polysaccharide rich biofilms on the surfaces studied here. However, in a review by Zheng et al. (2021), the influence of surface structures has been recently summarized showing that generally, bacteria tend to attach to surfaces more easily when they have a certain roughness, however there are also some rough surfaces achieving the opposite and also different bacterial species can react in different ways to the same surface.

Biofilm gene regulation did not show particularly big differences between the compared groups. As no RNA*later*^®^ or comparable reagent was used to freeze the transcriptome after biofilm removal a shift in the transcriptome might have occurred during the biofilm processing steps. However, as all samples were treated in the same way the relative impact is expected to be the same on the individual samples. For *S. aureus*, Resch et al. (2005) reported the genes *icaA* and *icaC*, which are commonly involved in early biofilm development, not to be upregulated in biofilms compared to planktonic growth after 48 h of growth. Expression of *icaA*, *icaC*, and *aap* gene regulation in *S. epidermidis* biofilms on different materials was reported by Patel et al. (2012) still to be active after 48 h of biofilm development. In an earlier study we found that on different Titanium-alloy surfaces the gene expression of the same set of genes of *S. aureus* Newman D2C followed a similar trend (Paulitsch-Fuchs et al., 2021) in that if there was a statistical difference in the regulation it was a decrease compared to the control (which was the untreated TiAl6V4 alloy, a rather smooth surface). Atshan et al. (2013) reported for four different MRSA isolates peak values of *fnbA*, *fnbB*, and *clfB* after 24 h. Comparing this with our results we conclude that the measurement point at 48 h is late into biofilm development and therefore no upregulation of the genes could be detected (with exception of fnbB on CoCrMo TiN). Together, the results point in the direction that the biofilm at 48 h is already well established and it is possibly not necessary that genes of early attachment are still upregulated. There is no difference in gene regulation between smooth and rough surfaces detectable at this timepoint. The *S. epidermidis* strain used in this study is *icaA* and *icaC* gene negative (this has been confirmed during this study; data not shown). As those genes are involved in the biosynthesis machinery necessary for PIA production in staphylococcal biofilms it is interesting that polysaccharides have been detected in the *S. epidermidis* strain which is *ica* negative. The explanation might be that other polysaccharide species (which are not regulated by the *ica* family of genes) are expressed in the strain used in this study. For example Spiliopoulou et al. (2012), have reported a 20-kDa polysaccharide (composed of glucose and N-acetylglucosamine) in the *S. epidermidis* EPS which is independent from *ica*. The two genes detected in the present study, *atlE* and *aap*, were both reported to be still active after 48 h of biofilm development by Patel et al. (2012). Similarly, the results presented here show an upregulation compared to the CoCrMo control on the smooth surfaces (not significant), the biofilms on the rough surfaces show a downregulation in at least one of the two genes after 48 h, CoCrMo cpTi in both genes (one of them significantly lower than the control). On those rough surfaces, the amount of proteins and polysaccharides was significantly higher in all cases, indicating that single cells in biofilms on rougher surfaces are producing a higher amount of proteins and polysaccharides. Otto (2013) shows a difference between early and late maturation phase in terms of adhesive and disruptive factors. We speculate that the biofilms on the rough surfaces in our study do not need the *atlE* and *aap* gene products, which are involved mainly in mediation of the early attachment and in accumulation (Patel et al., 2012; Arciola et al., 2015), anymore. To prove this conclusively further studies on biofilm developmental stages are necessary.

In conclusion, the rough CoCrMo surface-modifications are prone to biofilms showing a higher amount of proteins and polysaccharides. The transcription rate of the genes studied here needs to be studied at different time points in order to draw a hard conclusion as to the impact of surfaces on the regulation of those genes. Follow-up studies therefore should include more time points, defined biofilm forming strains and clinical isolates for gene analysis in order to get a better understanding of time-dependent development. In addition, a study of human osteoblast cell cultures and bacterial cells being co-incubated on the surfaces might provide insights in the competition for the place on the surfaces.

## Data Availability Statement

The original contributions presented in the study are included in the article/supplementary material. Further inquiries can be directed to the corresponding author.

## Author Contributions

A.P-F.: Conceptualization, Methodology, Validation, Visualization, Supervision, Writing—Original Draft, Writing—Review & Editing, Project administration. B.B.: Investigation, Formal analysis, Writing Original Draft. L.W.: Investigation, Formal analysis, Visualization. N.D.: Statistical Assessment, Rewriting and Editing. N.E.: Investigation, Formal Analysis. B.L.: Funding acquisition, Project administration, Supervision, Writing—Review & Editing. All authors listed have made a substantial, direct, and intellectual contribution to the work and approved it for publication.

## Funding

The authors acknowledge financial support from the FFG Bridge program (Grant No. 861608).

## Conflict of Interest

The authors declare that the research was conducted in the absence of any commercial or financial relationships that could be construed as a potential conflict of interest.

## Publisher’s Note

All claims expressed in this article are solely those of the authors and do not necessarily represent those of their affiliated organizations, or those of the publisher, the editors and the reviewers. Any product that may be evaluated in this article, or claim that may be made by its manufacturer, is not guaranteed or endorsed by the publisher.
